# The tankyrase-specific inhibitor JW74 affects cell cycle progression and induces apoptosis and differentiation in osteosarcoma cell lines

**DOI:** 10.1002/cam4.170

**Published:** 2013-12-17

**Authors:** Eva Wessel Stratford, Jeanette Daffinrud, Else Munthe, Russell Castro, Jo Waaler, Stefan Krauss, Ola Myklebost

**Affiliations:** 1 Cancer Stem Cell Innovation Centre and Department of Tumor Biology, Institute of Cancer Research, The Norwegian Radium Hospital, Oslo University HospitalPO Box 4953 Nydalen, Oslo, NO-0424, Norway; 2 Department of Molecular Bioscience, University of OsloPO Box 1041 Blindern, Oslo, NO-0316, Norway; 3 SFI-CAST Biomedical Innovation Center, Unit for Cell Signaling, Oslo University HospitalForskningsparken, Gaustadalleén 21, Oslo, 0349, Norway

**Keywords:** *β*-catenin, Differentiation, JW74, *let-7*, osteosarcoma, tankyrase, TNKS inhibitior, Wnt

## Abstract

Wnt/*β*-catenin is a major regulator of stem cell self-renewal and differentiation and this signaling pathway is aberrantly activated in a several cancers, including osteosarcoma (OS). Attenuation of Wnt/*β*-catenin activity by tankyrase inhibitors is an appealing strategy in treatment of OS. The efficacy of the tankyrase inhibitor JW74 was evaluated in three OS cell lines (KPD, U2OS, and SaOS-2) both at the molecular and functional level. At the molecular level, JW74 induces stabilization of AXIN2, a key component of the *β*-catenin destruction complex, resulting in reduced levels of nuclear *β*-catenin. At the functional level, JW74 induces reduced cell growth in all three tested cell lines, in part due to a delay in cell cycle progression and in part due to an induction of caspase-3-mediated apoptosis. Furthermore, JW74 induces differentiation in U2OS cells, which under standard conditions are resistant to osteogenic differentiation. JW74 also enhances differentiation of OS cell lines, which do not harbor a differentiation block. Interestingly, microRNAs (miRNAs) of the *let-7* family, which are known tumor suppressors and inducers of differentiation, are significantly upregulated following treatment with JW74. We demonstrate for the first time that tankyrase inhibition triggers reduced cell growth and differentiation of OS cells. This may in part be due to an induction of *let-7* miRNA. The presented data open for novel therapeutic strategies in the treatment of malignant OS.

## Introduction

Wnt/*β*-catenin signaling is involved in multiple biological processes, including regulation of cellular proliferation and the switch between stem cell–ness and differentiation 1–4. Altered Wnt/*β*-catenin signaling has been linked to degenerative diseases, metabolic diseases, and cancer 2,5–7. The key mediator of canonical Wnt signaling, *β*-catenin, is found at multiple subcellular localizations, including adherence junctions where it contributes to stabilizing cell–cell contacts, and in the nucleus where *β*-catenin is involved in transcriptional regulation 2,4,8.

The Wnt/*β*-catenin signaling pathway is activated when Wnt ligand binds to Frizzled (FZD) receptors and low-density lipoprotein receptor-related proteins-5/6 (LRP5/6) coreceptors. As a result, *β*-catenin accumulates in the cytoplasm and subsequently translocates to the nucleus where it regulates transcription of Wnt/*β*-catenin target genes, in part by binding to transcription factor T-cell factor/lymphoid enhancer-binding factor (TCF/LEF) 6.

In the absence of Wnt signaling, *β*-catenin levels are tightly controlled by the cytoplasmic destruction complex (DC), which consists of the rate-limiting proteins AXIN1/2, the adenomatous polyposis coli protein (APC), casein kinase (CK1)*α,* and glycogen synthase kinase 3 (GSK3)*β* and additional associated proteins including TRF-1-interacting ankyrin-related ADP-ribose polymerase 1 or 2 (tankyrase 1/2; TNKS1/2; ARTD5/6) 4,9. *β*-catenin associates with the DC, is phosphorylated by CK1-*α* and GSK3*β* 10–12, and subsequently ubiquitinated and degraded 13,14. Recently, it was shown that TNKS, at least in part, regulates this process through poly (ADP ribosyl)ating AXIN and itself, as well as the ubiquitin ligase RNF146, a process that initiates ubiquitination and degradation 15–18. Thus, through the control of the stability of the rate-limiting DC protein AXIN1/2, *β*-catenin levels can be attenuated by TNKS 19.

Due to the biological relevance of Wnt/*β*-catenin signaling, considerable efforts have been made to identify drugs that inhibit Wnt/*β*-catenin signaling either by blocking Wnt secretion 20 or by interfering with *β*-catenin binding to its transcription factor targets 4,7,16,17,20,21. Recently, drugs which block the catalytic PARP domain of TNKS1/2 (XAV939, IWR-1, JW55, JW74, G007-LK, WIKI4) have been identified and shown to inhibit Wnt/*β*-catenin signaling 16,17,20–23.

Osteosarcoma (OS) is the most common primary malignant bone cancer 24 and although the majority of patients undergo an aggressive treatment regime, often including surgery, radiotherapy, and chemotherapy, prognosis remains poor 25. OS is characterized by the presence of abnormal osteoblasts. Thus, imbalance in the osteogenic differentiation process is central to the disease, and in agreement with this, more than 80% of OS tumors are poorly differentiated and of higher grade 26. Wnt/*β*-catenin signaling is implicated in normal osteoblast differentiation and aberrant Wnt/*β*-catenin signaling disrupts normal bone development 6 and is frequently observed in OS 27. Mutations in *β*-catenin have not been observed in OS, but instead increased *β*-catenin activity has been linked to increased expression of Wnt receptors or an inhibition or loss of expression of secreted inhibitors 28. Indeed, elevated expression of the receptor LRP5 was observed in 50% of high-grade OS tumors and expression correlated with metastasis 29. Inhibition or loss of expression of the secreted inhibitor Wnt inhibitory factor (WIF1) was observed in 76% of OS patient samples in a different study 30,31. As elevated Wnt signaling is a common event in OS, inhibitors of Wnt/*β*-catenin may have therapeutic potential for OS patients 28. In this study, we have investigated the effect of the tankyrase-specific inhibitor JW74 on OS cell lines KPD, U2OS, and SaOS-2 at the molecular and functional level.

## Materials and Methods

### Cell lines, culture conditions, and reagents

The cell lines U2OS, SaOS-2 (both from American type culture collection [ATCC]), and KPD 32 were cultured in RPMI-1640 (Life Technologies, Carlsbad, CA) supplemented with 10% fetal bovine serum (FBS) (PAA laboratories Gmbh, Pashing, Austria), glutamax, and penicillin/streptomycin (both from Life Technologies). Short tandem repeat (STR)-DNA profiling of 15 loci and amelogenin was performed (Genetica DNA Laboratories, Cincinnati, OH) and U2OS and SaOS-2 profiles were validated by comparing to the ATCC database. The KPD STR-DNA profile was validated by matching the obtained profile with a profile from a xenograft, generated from the original patient sample. JW74 21 was dissolved in dimethyl sulfoxide (DMSO) (10 mmol/L) and stored at 4°C for maximum 2 weeks. Dilutions in culturing medium to final concentrations of 10–0.5 *μ*mol/L were done immediately before use.

### Western blotting

One hundred fifty thousand cells grown overnight in six-well plates were treated with 0.1% DMSO (control) or JW74 (10–0.5 *μ*mol/L) for 24, 48, or 72 h. Cell lysates were generated by incubating in 200 mL lysis buffer (5 mol/L NaCl, 0.5 mol/L Tris-base, NP-40, and protease and phosphatase inhibitors) on ice for 10 min, followed by a short sonication. Proteins were separated by sodium dodecyl sulfate polyacrylamide gel electrophoresis (SDS-PAGE) and immunoblotting was performed using primary antibodies; AXIN2 (76G6) (Cell Signaling Technology, Boston, MA), Tankyrase-1/2 (H-350) (Santa Cruz Biotechnology, Dallas, TX), LaminB1 (Abcam, Cambridge, UK), active *β*-catenin ABC (Millipore, Billerica, MA), total *β*-catenin (610154) (BD Transduction Laboratories™, Franklin Lakes, NJ), and ACTIN (Santa Cruz Biotechnology). Antibodies were visualized using secondary horseradish peroxidase-conjugated antibodies (P0260, P0448 or P0449, DakoCytomation, Glostrup, Denmark) and enhanced chemiluminescent substrate (SuperSignal West Dura extended duration substrate; Thermo Scientific, Waltham, MA).

### Reporter luciferase assay

Transfection of 2000 U2OS cells plated in 96-well plates was done the following day with reporter plasmid pTA-Luc-STF and control plasmid-expressing Renilla, as in 21. Transfected cells were incubated for 48 h in culturing media supplemented with 0.1% DMSO (control) or JW74 (1 *μ*–10 *μ*mol/L). Luciferase and Renilla activity were determined using Dual-Glo Luciferase Assay System (Promega, Madison, WI).

### Quantitative real-time polymerase chain reaction

Isolation of total RNA and cDNA synthesis were performed using Cell-to-Ct kit for mRNA or miRNA (Ambion, Austin, TX), following the manufacturer's protocol. Quantitative real-time polymerase chain reaction (qRT-PCR) was performed with primers and master mix from Ambion, using cDNA from 100 to 500 cells/well. The detection limit was set to cycle threshold value = 36. Relative quantifications were calculated with the 2^−ΔΔCt^ method normalizing to *PGK1* or *RNU44* for mRNA and miRNA analyses, respectively. *PGK1* or *RNU44* were used as housekeeping genes, due to their unchanged expression during treatment 33. Data were presented relative to the DMSO-treated sample.

### Cell cycle analyses

Three hundred thousand cells in T25 flasks were attached overnight and treated for 72 h with DMSO (control) or 5 *μ*mol/L JW74. Two million treated cells were stained with 2 *μ*g/mL Hoechst 33342 and 20 *μ*L/test of PE-mouse anti-human Ki-67 (BD Pharmigen, San Diego, CA), as described previously 34. Flow cytometric analyses were performed using Becton Dickinson LSRII Flow Cytometer. Minimum 100,000 cells were acquired per sample, and gating on forward scatter versus side scatter was used to exclude cell debris and doublets. Data analysis was performed using FlowJo (TreeStar, Inc., Ashland, OR).

### Proliferation assay

Two to three thousand cells attached overnight in 96-well plates were treated with culturing medium containing 0.1% DMSO (control) or JW74 (10–0.1 *μ*mol/L). Proliferation rates based on cell confluence were determined by live cell imaging (IncuCyte; Essens Bioscience, Birmingham, U.K.), as described previously 35. Cellular viability was also determined by MTS assay (3-[4,5-dimethylthiazol-2-yl]-5-[3-carboxymethoxyphenyl]-2-[4-sulfophenyl]-2H-tetrazolium) (Promega), according to the manufacturer's protocol. Expression of the proliferation marker Ki-67 was performed by staining cells with PE-mouse anti-human Ki-67 (BD Pharmigen) and by analyzing the expression by flow cytometry, as described earlier.

### Apoptosis assay

For Caspase-3 assay, cells were plated and treated as for the proliferation assay. In addition, Cell player reagent (5 mmol/L in DMSO) (Essens Bioscience) was included in the medium (1:1000 dilution), enabling quantitative measurement of Caspase-3 activity by fluorescence live cell imaging in the IncuCyte. Data show total number of cells with high Caspase-3 activity in each well 52 h post treatment-start. Annexin V assay was performed using the Alexa Fluor 488 annexin V and propidium iodide (PI) kit for flow cytometry (Invitrogen, Carlsbad, CA). 100,000 U2OS cells were plated in six-well plates and incubated with DMSO or 10 *μ*mol/L JW74 for 72 h and subsequently analyzed according to the protocol provided by the manufacturer. In brief, Alexa 488-labeled Annexin V binds to phosphatidyl serines exposed on the outer leaflet of the plasma membrane of apoptotic cells. PI was used to exclude necrotic cells from the assay.

### Osteogenic differentiation and quantitative and qualitative assessment of the process

Thirty thousand cells attached overnight in 24-well plates were incubated in culturing medium supplemented with one of four combinations: (1) 0.1% DMSO (control); (2) JW74 (10 *μ*mol/L) only; (3) 0.1% DMSO in combination with a differentiation cocktail (10 mmol/L glycerol phosphate, 10 nmol/L dexamethasone, and 50 *μ*g/mL ascorbic Acid), or (4) differentiation cocktail combined with JW74 (10 *μ*mol/L). Cells were not passaged during the experiment (maximum 24 days), but medium and supplements were changed twice per week. Osteogenic differentiation was determined quantitatively, using alkaline phosphatase (ALP) activity as a marker. The ALP assay kit (Abcam) was performed as recommended by manufacturer. Data are presented relative to total protein concentration. Degree of osteogenic differentiation was also assessed by alizarin red staining (40 mmol/L alizarin red S solution for 20 min).

### Statistical analyses

Statistical analyses were performed using SigmaPlot 11 (Systat Software Inc., Chicago, IL). For comparisons of two groups, normal distributions of datasets were first analyzed with the Shapiro–Wilk test. When the Shapiro–Wilk test passed (*P* = > 0.05), Student's *t*-test was performed. If the Shapiro–Wilk test failed (*P* < 0.05), Mann–Whitney rank sum test was applied. *P* < 0.05 was regarded as a statistically significant difference.

## Results

### The tankyrase inhibitor JW74 reduces *β*-catenin levels in OS cell lines

We selected three OS cell lines for testing the efficacy of the tankyrase-specific inhibitor JW74. U2OS and SaOS-2 were chosen due to increased expression of LRP5 receptor and several isoforms of the FZD receptor 29, as well as reduced expression of WIF1 30,31, resulting in aberrant activation of Wnt/*β*-catenin signaling. With regard to differentiation status, SaOS-2 is considered more differentiated, consistent with high-basal ALP activity 36. On the contrary, U2OS is more undifferentiated, with resistance to undergo in vitro osteogenic differentiation, consistent with low and noninducible basal ALP levels 36,37. Thus, the two cell lines enabled us to study the efficacy of Wnt/*β*-catenin inhibition in opposing differentiation contexts. From a panel of well-characterized OS cell lines 38, we also included KPD, which is a less well-studied cell line in the context of Wnt/*β*-catenin signaling, but like U2OS and SaOS-2, was reported to express increased *AXIN2* mRNA levels 39.

**Figure 1:**
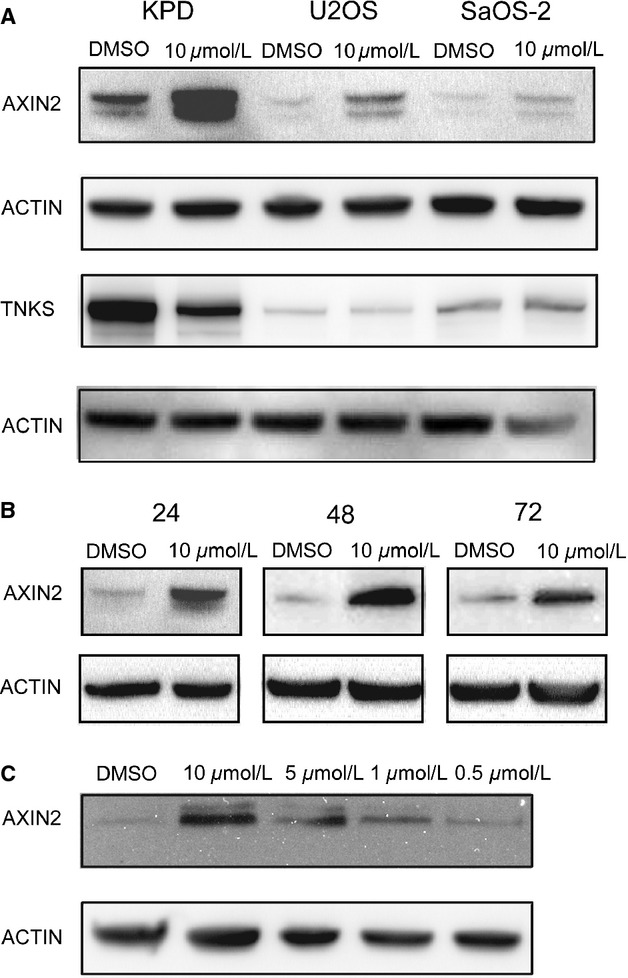
Effects of JW74 treatment on AXIN2 and TNKS protein levels in OS cells. (A) Total cell lysates from KPD, U2OS, or SaOS-2 cells extracted following 72 h treatment with 0.1% DMSO (control) or 10 *μ*mol/L JW74 were analyzed by Western blotting using antibodies against AXIN2, TNKS1/2, and ACTIN (loading control). (B) U2OS total cell lysates generated following 24, 48, or 72 h treatment with 10 *μ*mol/L JW74 or 0.1% DMSO (control) were analyzed by Western blotting, showing that AXIN2 protein levels are elevated by 24 h and remain so 48 and 72 h following drug treatment. (C) U2OS cells were treated with 0.1% DMSO (control) or JW74 (0.5–10 *μ*mol/L) for 48 h, demonstrating dose-response stabilization of AXIN2. OS, osteosarcoma.

**Figure 2:**
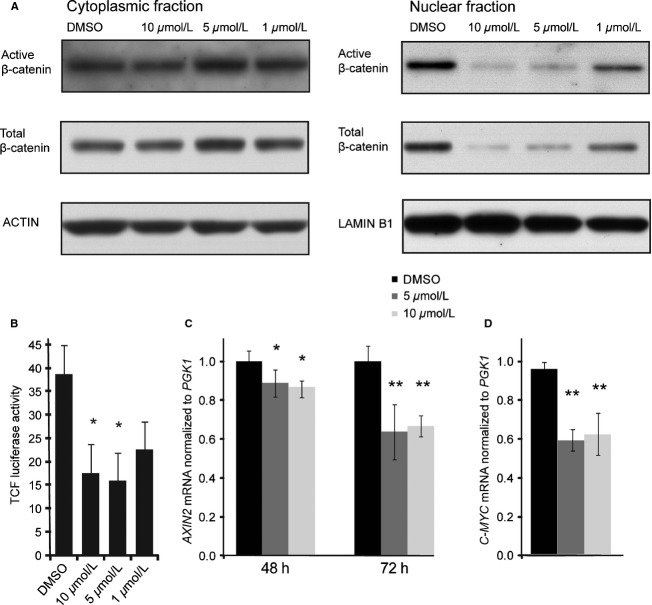
JW74 treatment reduces nuclear active *β*-catenin levels and inhibits transcription of downstream targets. (A) Cytoplasmic and nuclear fractions extracted from U2OS cells following 48 h treatment with 0.1% DMSO (control) or 10 *μ*mol/L JW74 were analyzed by Western blotting using antibodies against active *β*-catenin, total *β*-catenin, ACTIN, or LAMINB1 (loading controls). (B) TCF/LEF reporter assays demonstrate that JW74 inhibits *β*-catenin mediated activity in U2OS cells. Cells transfected with pTA-Luc-STF and Renilla plasmids were treated with 0.1% DMSO (control) or JW74 (0.1–10 *μ*mol/L) for 48 h. Data are normalized to Renilla. Significantly decreased reporter activity was observed following treatment with 10 *μ*mol/L JW74 (*P = 0.033) and 5 *μ*mol/L JW74 (*P = 0.024). (C) *AXIN2* mRNA levels were significantly reduced following JW74 treatments of U2OS cells for 48 h (*5 *μ*mol/L JW74: *P* = 0.005 and 10 *μ*mol/L JW74: *P* = 0.042) and 72 h (**5 *μ*mol/L and 10 *μ*mol/L *P* < 0.001). (D) *C-MYC* mRNA levels were significantly reduced following incubation of U2OS cells for 48 h (**5 *μ*mol/L and 10 *μ*mol/L *P* < 0.001). Analyses were performed by qRT-PCR and presented data are normalized to *PGK1* and relative to DMSO-treated samples. Error bars represent standard deviation. qRT-PCR, quantitative real-time polymerase chain reaction. TCF/LEF, T-cell factor/lymphoid enhancer-binding factor.

Following treatment with JW74, stabilization of AXIN2 was demonstrated in all three OS cell lines by Western blotting (Fig. 1A). AXIN2 stabilization is considered a reliable marker of tankyrase inhibition in the context of the DC 16,17,40. We also wanted to determine the TNKS1/2 protein levels in the three cell lines following JW74 treatment, as TNKS1/2 protein levels can be either stabilized or destabilized in response to tankyrase inhibition, depending on context 40. Alterations in TNKS1/2 protein levels after JW74 treatment were varied in the OS cell lines (Fig. 1A). While KPD cells displayed a clear reduction in TNKS, TNKS levels were unaltered in U2OS cells, and in SaOS-2 cells we observed slightly increased TNKS levels (confirmed by quantification of TNKS1/2 relative to ACTIN). The drug response was sustained, as AXIN2 protein levels were strongly elevated at 24 h, and remained increased throughout 72 h incubation with 10 *μ*mol/L JW74 (Fig. 1B). AXIN2 stabilization was dose-dependent, being in U2OS cells effective across the range from 1 to 10 *μ*mol/L JW74 (Fig. 1C, confirmed by quantification). Although AXIN2 stabilization did not alter cytoplasmic *β*-catenin levels in these cells as measured by Western blot, nuclear levels of total *β*-catenin and active *β*-catenin (also known as ABC) were strongly reduced in a dose-dependent manner (Fig. 2A). The reduction in nuclear *β*-catenin translated into reduced transcriptional activity of a TCF/LEF-based luciferase reporter (Fig. 2B). Accordingly, transcription of the *β*-catenin target gene *AXIN2* (Fig. 2C) and *C-MYC* (Fig. 2D) were reduced moderately, but significantly, following 48 and 72 h incubation with JW74.

### Tankyrase inhibition reduces growth, increases apoptosis, and delays cell cycle progression

**Figure 3:**
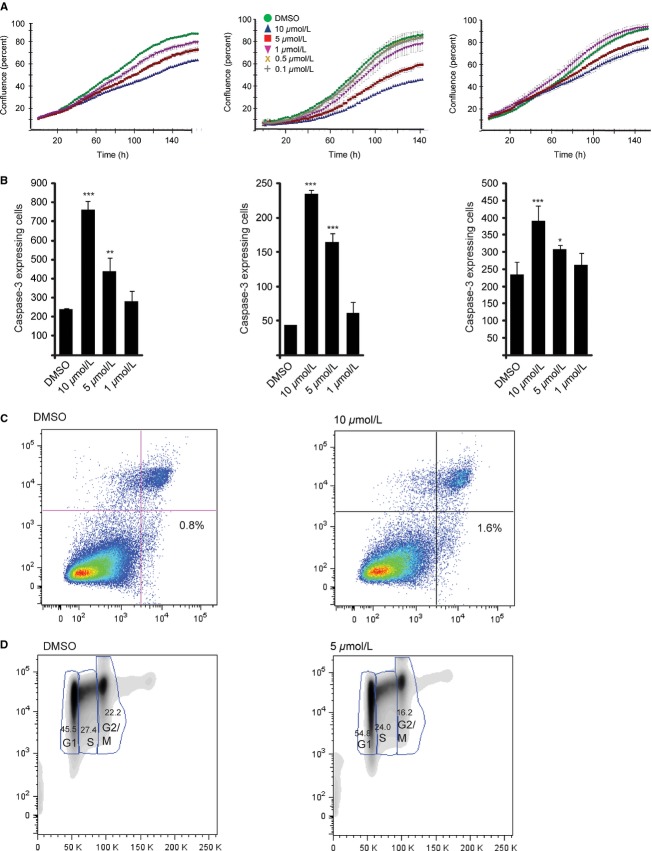
JW74 treatment inhibits osteosarcoma (OS) growth. (A) The proliferative capacity of KPD, U2OS and SaOS-2 was inhibited following treatment with JW74 (1–10 *μ*mol/L). Cell densities were measured by IncuCyte live cell imaging. DMSO was included as control. (B) The number of Caspase-3-expressing cells per well, following 52 h exposure to drug was determined using the IncuCyte live cell imaging system. Caspase-3 activity was significantly increased in a dose-dependent manner (*P = 0.014; **P = 0.008; ***P < 0.001). Cells were treated as described in (A), including Cell player reagent in the culturing medium, which renders cells expressing increased levels Caspase-3 fluorescent. (C) The percentage of apoptotic U2OS cells increased from 0.8% (DMSO) to 1.6% (10 *μ*mol/L JW74) following 72 h drug treatment was determined by Alexa-488 Annexin V binding (*x*-axis). Propidium iodide (PI) was included as a marker of necrotic cells (*y*-axis). The analysis was performed by flow cytometry. A representative experiment is shown (D) JW74 treatment leads to accumulation of U2OS cells in G1 phase. The cells were treated with 0.1% DMSO (control) or 5 *μ*mol/L JW74 for 72 h and subsequently labeled with Hoechst (*x*-axis) and stained with proliferation marker Ki67 (*y*-axis). The number of cells in each cell cycle phase was determined by flow cytometry. A representative experiment is shown.

Having shown that JW74 exerts molecular effects on key mediators of the canonical Wnt signaling pathway, we next wanted to evaluate the functional effects of tankyrase inhibition. We first studied the proliferative capacity of OS cells during short-term in vitro treatment with JW74. For this purpose, we used the a live cell imaging machine (IncuCyte), which captures cellular images every second hour throughout the duration of the experiment enabling us to determine the effect of the drug on cell confluence over time. The time lapse experiment clearly showed that tankyrase inhibition had a dose-dependent growth-limiting effect on U2OS, KPD, and SaOS-2 cells (Fig. 3A). In addition to assessing proliferative capacity by live cell imaging, we tested the effect of tankyrase inhibition on cellular viability by performing an MTS assay and found that the cellular viability of U2OS cells treated for 72 h with 10 *μ*mol/L JW74 was reduced to 80%, relative to DMSO-treated cells (data not shown). We also performed flow cytometry to determined the expression of the proliferation marker Ki-67 in U2OS following 48 h treatment with DMSO or 10 *μ*mol/L JW74. Ki-67 expression was reduced from 97.5% in DMSO-treated cells to 86.7% in JW74-treated cells (data not shown).

We next used the live cell imaging machine to perform a Caspase-3 activity assay in U2OS, SaOS-2, and KPD cells treated with the tankyrase inhibitor. Interestingly, we found that Caspase-3 activity increased in a dose-dependent manner in all three cell lines (Fig. 3B). However, as others have shown that Caspase-3 was activated in several colon cancer cell lines, without resulting in the onset of apoptosis 41, we carefully examined serial images of individual Caspase-3-positive cells (appearing as green fluorescent). We observed membrane blebbing, detachment of the cells from the surface and production of apoptotic bodies and debris, morphological changes consistent with apoptosis. To investigate the onset of apoptosis by an additional method, we performed Annexin V flow cytometric analyses of U2OS cells treated with JW74 for 72 h. Also by this method, we observed increased apoptosis following drug treatment. The percentage of apoptotic cells bound by Alexa 488-Annexin V increased from 0.8% (DMSO) to 1.6% (10 *μ*mol/L) (Fig. 3C).

We subsequently performed flow cytometric cell cycle analyses of Hoechst-stained U2OS cells treated with 5 *μ*mol/L JW74 for 72 h and found an increased number of cells in the G1-phase (45.5–54.8%) and a decreased number of cells in S-phase (27.4–24.0%) and G2/M (22.2–16.2%) compared to control-treated cells (Fig. 3D), indicating that a delay in G1 contributes to the reduced growth rate. We did not observe any morphological changes indicative of senescence, such as flattened cellular morphology (data not shown). In agreement with these effects on the cell cycle, we observed significantly decreased expression of *CCND1* following exposure of U2OS cells to 5 *μ*mol/L JW74 for 48 h (∼twofold reduction; data not shown).

### Wnt/*β*-catenin inhibition induces osteogenic differentiation and leads to an increase in miRNAs of the *let-7* family

**Figure 4:**
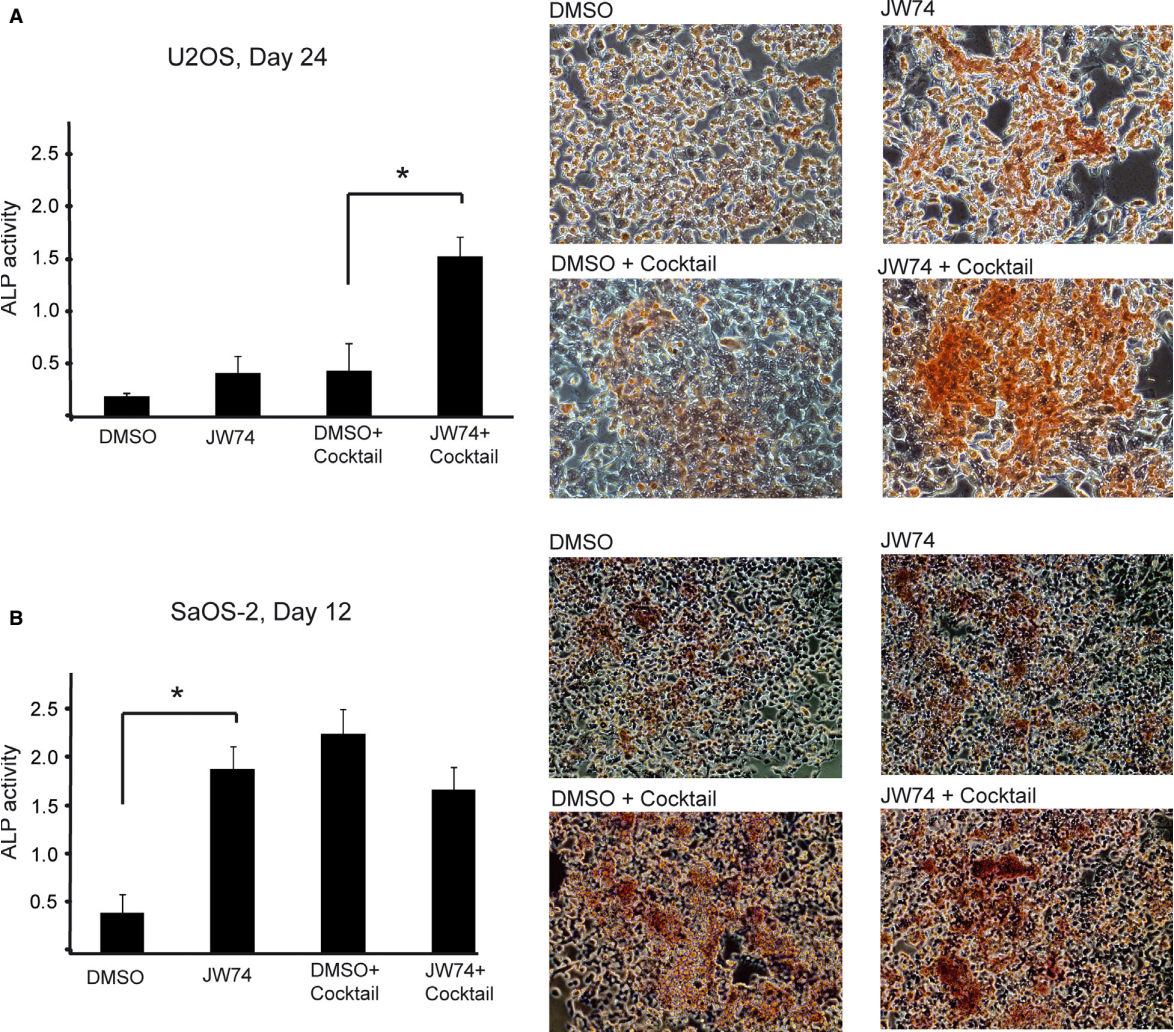
Long-term JW74 treatment induces cellular differentiation. Cells were treated as indicated, with either 0.1% DMSO only, 10 *μ*mol/L JW74 only, osteogenic differentiation cocktail combined with DMSO, or osteogenic differentiation cocktail combined with JW74 (10 *μ*mol/L). Quantitative measurements of ALP activity relative to total protein concentration and qualitative alizarin red staining are shown for (A) treated U2OS cells, day 24 and (B) treated SaOS-2 cells, day 12. Statistical significant differences in ALP levels are indicated by (*). Error bars represent standard deviation. ALP, alkaline phosphatase.

We subsequently went on to assess the effect of JW74 on differentiation. In agreement with previous studies, we found that U2OS cells did not spontaneously differentiate and showed only moderate signs of induced differentiation in the presence of osteogenic differentiation cocktail during a 24-day differentiation assay (Fig. 4A). This was determined quantitatively by measuring enzymatic ALP activity, an established osteogenic differentiation marker, and qualitatively by alizarin red staining, which marks calcium deposits generated in the mature osteoblasts on day 0, day 6, day 12, day 18, and day 24. Moderately increased ALP levels were observed in U2OS cells subjected to long-term incubation (24 days) with 10 *μ*mol/L JW74 alone, compared to control-treated cells (DMSO) (Fig. 4A). The changes were comparable to cells treated with differentiation cocktail, neither showing signs of full differentiation. However, when JW74 was combined with the differentiation cocktail, U2OS cells showed strong and unequivocal signs of differentiation, demonstrated by significantly increased ALP activity as well as alizarin red staining (Fig. 4A). We also observed that alizarin red-positive cells had morphological characteristics consistent with osteogenic differentiation, such as the presence of a small, round-celled body and long, thin processes (data not shown). Next, we investigated whether JW74 could improve the efficiency of differentiation in SaOS-2 cells. As expected, full differentiation was observed both qualitatively and quantitatively, when SaOS-2 cells were incubated with the standard differentiation cocktail for 12 days (Fig. 4B). Intriguingly, JW74 treatment alone induced differentiation in SaOS-2 cells equally efficient as differentiation cocktail and significantly better than cells treated with DMSO only. No additive effect was seen when differentiation cocktail was combined with JW74, presumably because maximal differentiation was already achieved.

**Figure 5:**
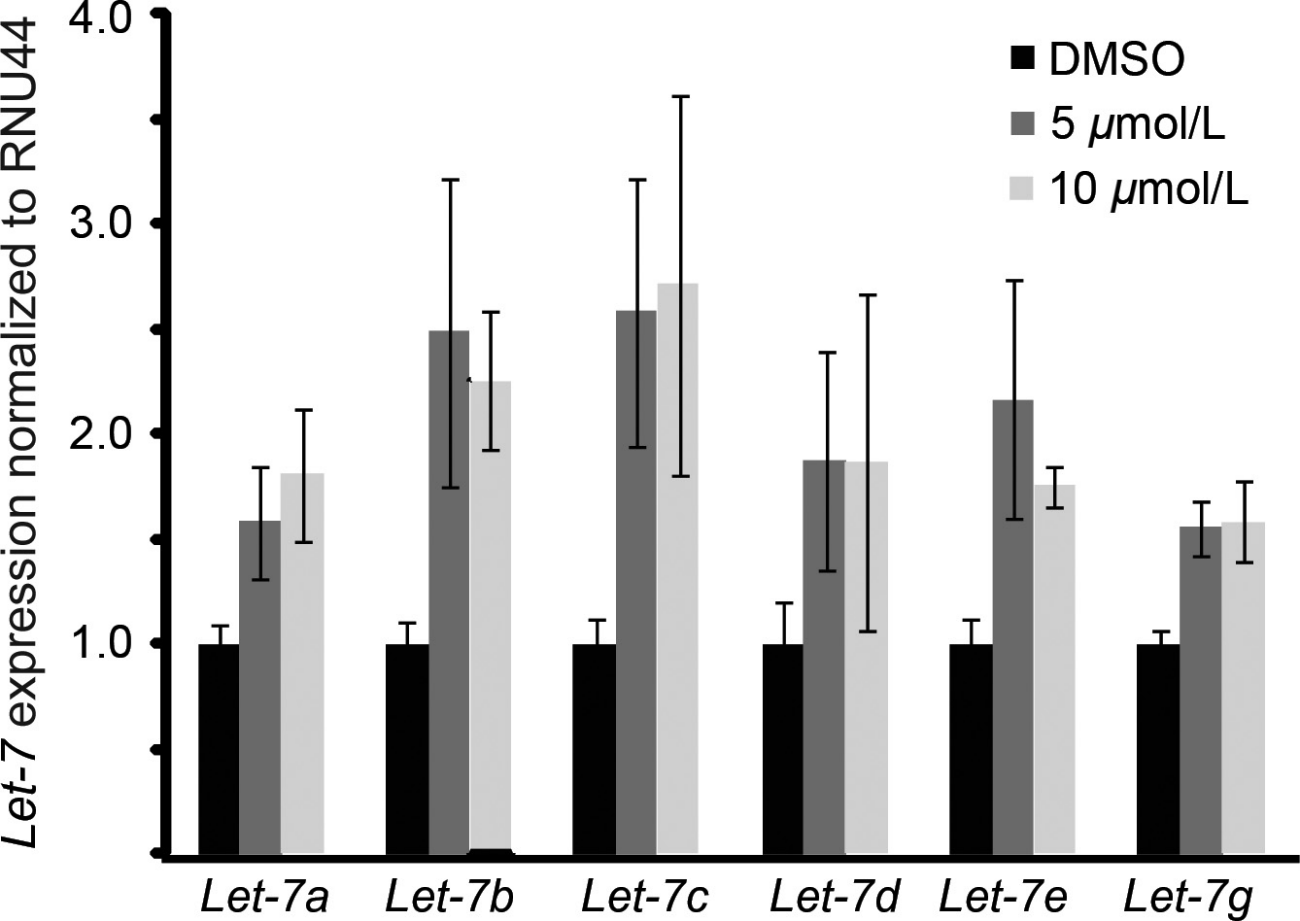
JW74 treatment leads to induction of *let-7* miRNA. qRT-PCR analyses demonstrating significantly increased (indicated by *) expression of *let-7* miRNA orthologs in U2OS cells treated 72 h with JW74 (5 or 10 *μ*mol/L). Data are normalized to RNU44 expression and relative to control-treated cells (DMSO). Error bars represent standard deviation. qRT-PCR, quantitative real-time polymerase chain reaction.

As JW74 treatment both induces osteogenic differentiation of OS cells and reduces *c-MYC* expression, we hypothesized that microRNA (miRNA) *let-7* levels might be elevated following JW74 treatment. miRNA *let-7* is a master regulator of differentiation 42, frequently reduced or lost in a range of cancers 43, and is negatively regulated by c-MYC. Indeed, we observed a solid increase in all the *let-7* orthologs evaluated (Fig. 5A) following 72-h treatment of U2OS cells with 5 or 10 *μ*mol/L JW74, as demonstrated by qRT-PCR analyses.

## Discussion

In this study, we present for the first time, the impact of tankyrase inhibition on representative OS cell lines using the novel specific tankyrase inhibitor JW74. In agreement with effects observed for colon cancer 16,17,20,21,40,44, we found that the TNKS-target AXIN2 was stabilized in all three OS lines evaluated. Furthermore, this resulted in reduced levels of *β*-catenin in the nucleus, reduced TCF/LEF reporter activity, and decreased *AXIN2* mRNA levels as demonstrated in U2OS cells. Similar to observations in treated colon cancer cell lines 17,21,40, TCF/LEF reporter activity was not lowered beyond 50%, indicating active feedback loops or alternative mechanisms preventing complete reduction in reporter activity. As TNKS, the primary drug target of JW74, is implicated in cellular functions beyond its role in the DC, such as telomere maintenance, glucose metabolism, and centrosome maturation 45, the observed effects may not be exclusively explained by altered *β*-catenin levels.

Functionally, OS cells treated with JW74 displayed reduced growth rate due to increased apoptosis and delayed cell cycle progression. This is consistent with the observed reduction in nuclear *β*-catenin levels and in agreement with findings in other cancer models 16,17,20,21,40,44, including synovial sarcoma 46. In addition, we found that tankyrase inhibition strongly induced differentiation of OS cells and enabled cells with resistance to induced differentiation to overcome their differentiation block.

The majority of OS tumors are poorly differentiated and induction of differentiation may be an interesting therapeutic strategy, as cells may become more susceptible to treatment upon induced differentiation 25. It has been suggested that OS should be considered a “differentiation disease” caused by genetic changes, which prevent full osteoblastic differentiation 47. The therapeutic potential of OS differentiation therapy has previously been demonstrated with nuclear receptor agonists, such as peroxisome proliferator-activated receptor (PPAR)*γ* agonists, which either on their own, or in combination with retinoids have been shown to inhibit proliferation, induce apoptosis, and most importantly, promote terminal differentiation of OS cells 48,49. Indeed, differentiation therapy with the retinoid all-trans retinoic acid is successfully used as standard treatment of acute promyelocytic leukemia patients 50. However, the observed differentiation induced by JW74 in this study did not correlate with an increase in *PPARγ mRNA* levels, following 72-h incubation with JW74 (data not shown).

It has also been shown that SOX2 plays a key role in maintaining OS cells in an undifferentiated state, being essential for self-renewal and acting as an antagonist of the Wnt pathway 51. However, JW74 treatment did not result in reduced *SOX2* expression in U2OS cells. Thus, mechanisms involving SOX2 do not seem responsible for the observed differentiation in our system.

The miRNA family *let-7* are tumor suppressors and key regulators of differentiation 42. Interestingly, we observed increased expression levels of multiple *let-7* orthologs following incubation with JW74. To our knowledge, neither tankyrase nor the Wnt/*β*-catenin signaling pathway has to date been directly linked with the *let-7* systems. As we observed reduced *C-MYC* levels following JW74 incubation, regulation of *let-7* through C-MYC is a possibility. However, further work is required to elucidate the links between tankyrase inhibition and increased *let-7* levels. Interestingly, *β*-catenin has been described as a regulator of other miRNAs, including miR-15, miR-16, miR-375, and miR-122a 52. However, the mechanisms through which *β*-catenin regulate these miRNAs are not known.

The significant upregulation of multiple *let-7* orthologs in response to JW74 treatment is of particular importance in the light of therapeutic attempts to reduce the proliferative capacity and trigger differentiation of poorly differentiated cancer cells through increased *let-7* levels. *Let-7* replacement therapy has shown great potential as a novel cancer therapeutic in xenograft models, where the tumor regresses following introduction of *let-7* 53–55. Our data suggest that similar therapeutic effects may be achievable by small drug inhibitors of tankyrase, establishing tankyrase as an important druggable biotarget, regulating a molecular switch between stem cell–ness and differentiation.
